# The Effect of Self-Esteem on Corrupt Intention: The Mediating Role of Materialism

**DOI:** 10.3389/fpsyg.2016.01063

**Published:** 2016-07-12

**Authors:** Yuan Liang, Li Liu, Xuyun Tan, Zhenwei Huang, Jianning Dang, Wenwen Zheng

**Affiliations:** Beijing Key Lab of Applied Experimental Psychology, School of Psychology, Beijing Normal UniversityBeijing, China

**Keywords:** self-esteem, corrupt intention, materialism, unethical behavior, psychological process

## Abstract

The present set of studies aimed to explore the effect of self-esteem on corrupt intention and the mediating role of materialism in generating this effect. In Study 1, we used questionnaires to investigate the correlation among self-esteem, materialism, and corrupt intention. In Study 2, we manipulated self-esteem to explore the causal effect of self-esteem on materialism and corrupt intention. In Study 3, we manipulated materialism to examine whether inducing materialism can reduce the relationship between self-esteem and corrupt intention. The three studies converged to show that increased self-esteem caused a low level of materialism, which in turn decreased corrupt intention. The theoretical and practical implications of the results are discussed.

## Introduction

Corruption refers to the misuse of public power for private gain (Treisman, 2000). It has brought great negative influences on our society. For instance, corruption increases the cost of doing business by up to 10% on average, and decreases 5% investment in corruptive countries than that in relatively corruption-free countries. It is estimated that the annual cost of corruption worldwide is US$2.6 trillion with over US$1 trillion paid in bribes each year (Organization for Economic Co-operation and Development [OECD], 2014). In addition, citizens turning out at elections in very corrupt countries are 20–30% fewer compared with countries with little corruption (Stockemer et al., 2013). Given that such heavy cost is hard to afford, it is important to figure out the psychological underpinnings of corruption, and thereby to provide scientific support for fighting against corruption.

Corruption has recently received greater attention from psychologists. The subsequent research has identified important antecedents of corruption such as socio-economic status (Olken, 2009; Charron, 2016), cultural orientation (Li et al., 2006; Mazar and Aggarwal, 2011; Huang et al., 2015), risk attitude (Berninghaus et al., 2013), and social dominance orientation (Tan et al., 2016b). Inspired by the ideas that self-esteem could be maintained and enhanced by material possessions or prestige (Sivanathan and Pettit, 2010; Jiang et al., 2015), and that corruption offers a rapid way to obtain admirable status and material resources (You, 2007; Johnston, 2012), the current research investigates an issue that has been overlooked: the effect of self-esteem on corruption. Namely, it aims to address two questions: Does self-esteem influence corrupt intention? If so, what is the underlying psychological process of the association?

### Self-Esteem and Corrupt Intention

Self-esteem refers to the overall self-evaluation of one’s worth, and it is a universal and fundamental human need (Allport, 1955; Maslow, 1968; Taylor and Brown, 1988; Solomon et al., 1991; Baumeister et al., 1993). Most individuals aim to protect, maintain, and enhance their self-esteem (Baumeister, 1998). Individuals with high self-esteem believe they can succeed and enhance themselves based on their own merits and are less concerned with avoiding failure (Blaine and Crocker, 1993; Vohs and Heatherton, 2001). By contrast, individuals with low self-esteem feel inferior, unworthy, lonely, insecure, anxious and depressed, uncertain about themselves, and particularly challenged to succeed, and they interpret events and feedback in terms of what they indicate about their self (Mruk, 1995; Brown and Marshall, 2001; Pyszczynski et al., 2004; Donnellan et al., 2005). According to Self-Affirmation Theory (Steele, 1988), when people feel uncertain in one domain, they compensate for this by “spontaneously emphasizing certainty and conviction about unrelated attitudes, values, personal goals, and identifications” (McGregor et al., 2001, p. 473). This compensation constitutes part of a hydraulic motivational process called compensatory conviction. Therefore, individuals whose self-esteem is threatened are motivated to seek any boost to compensate for low self-esteem (Crocker and Park, 2004).

Achieving rewards and status could facilitate self-affirmation and likewise enhance self-esteem for individuals lacking it (Sivanathan and Pettit, 2010). Many unethical behaviors, such as corruption, can be performed to facilitate such achievement. Corruption, like other dishonest acts, is not only motivated by external benefits, but also by internal rewards (Mazar et al., 2008). In order to enhance self-esteem, individuals with low self-esteem divert their attention from fulfilling intrinsic fundamental human needs to pursuing extrinsic outcomes, which pursuit exacerbates already poor self-regulation (Baumeister and Leary, 1995; Deci and Ryan, 2000; Crocker, 2002). Thus, these individuals are more likely to adopt risky, self-aggrandizing, get-rich-quick schemes (Tice, 1993; Zywica and Danowski, 2008) to secure admiration. Corruption offers a rapid way to obtain admirable status and material resources (You, 2007; Johnston, 2012), and even though these items are not theirs (Ledeneva, 1998), they can enhance self-esteem (Richins and Dawson, 1992; Chang and Arkin, 2002; Wattanasuwan, 2005; Isaksen and Roper, 2012). Therefore, individuals with depressed self-esteem prefer to use corruption as a crutch to enhance their self-worth. By contrast, positive self-esteem is not in desire for enhancement, which leads individuals to adhere to ethical standards rather than engage in corruption (Aronson and Mettee, 1968; Tice, 1993; Mesmer-Magnus et al., 2010; Barkan et al., 2015; Jordan et al., 2015). Thus, we hypothesize that *high self-esteem decreases corrupt intention (Hypothesis 1).*

### Materialism as a Mediator

If one is increasingly driven by extrinsic goals such as wealth, possessions, image, and status to affirm the self and to seek compensation for poor self-esteem, one might be mired by materialism. Materialism refers to the elevated importance placed by a person on possessions and their acquisition as a necessary or a desirable means of attaining desired end states, including happiness (Richins and Dawson, 1992). Research has indicated that materialism can be used to compensate for threatened self-esteem (Shrum et al., 2013; Jiang et al., 2015) and may prompt unethical behavior (Kouchaki et al., 2013; Gino and Mogilner, 2014). It is thus reasonable to assume that materialism might mediate the effect of self-esteem on corrupt intention. If materialism accounts for the effect of self-esteem on corrupt intention, then we can block materialism to control corruption in individuals with low self-esteem.

Indeed, the earliest sophisticated attempt to measure materialism (Belk, 1985) conceived of the construct as a trait, and more recent theoretical statements have proposed that materialism is an aspect of identity (Dittmar, 2008; Shrum et al., 2013). By contrast, following Richins and Dawson (1992), the current set of studies regards materialism as value or a goal that reflects the extent to which an individual believes acquiring money or possessions is important. It also conveys the striving for the related objects of an appealing image and a high status/popularity, both of which objects are frequently expressed through money and possessions (Kasser, 2016). Understanding materialism as a value/goal allows us to test hypotheses about both a person’s relatively stable disposition toward materialism and what occurs when materialistic values/goals are momentarily activated in a person’s mind.

It has been shown that self-esteem is negatively associated with materialism (Isaksen and Roper, 2012; Kasser et al., 2014). Self-esteem helps individuals respond to self-worth threats by emphasizing their competence or dominance and become more independent (Blaine and Crocker, 1993; Vohs and Heatherton, 2001). Individuals with high self-esteem do not require many material possessions for purposes of gaining a certain status, image, admiration (Richins and Dawson, 1992), obtaining ephemeral economic safety (Christopher et al., 2006; Clark et al., 2011), restoring psychological security (Noguti and Bokeyar, 2014), affirming one’s self-identity (Chang and Arkin, 2002; Wattanasuwan, 2005), or coping with doubts concerning self-worth or competence (Chang and Arkin, 2002; Kasser, 2002; Jiang et al., 2015). Individuals with low self-esteem are in contrast likely to use more money to compensate for their impaired self-esteem (Shrum et al., 2013; Jiang et al., 2015) and to require prestige and many possessions to identify themselves (Richins and Dawson, 1992; Magee and Galinsky, 2008; Mogilner and Aaker, 2009). This necessity thus generates a powerful inner drive to acquire many impressive belongings. This pattern implies that high self-esteem might decrease materialism.

Existing literature indicates that materialism is positively associated with unethical behaviors (Tang and Chiu, 2003; Tang et al., 2008; Tang and Liu, 2011). Simply primed with money leads individuals to be less helpful and less fair when they interact with others, work harder toward their personal goals (Vohs et al., 2006, 2008; Zhou et al., 2009; Yang et al., 2013; Gino and Mogilner, 2014), and even engage in unethical behaviors (Kouchaki et al., 2013). Empirical evidence indicates that individuals with high levels of materialism are more self-oriented; more focused on wealth, achievement, power, and status; and less concerned with others (Richins and Dawson, 1992; Schwartz, 1992; Sheldon and McGregor, 2000; Bauer et al., 2012; Gino and Mogilner, 2014). Materialism has been associated with consumers actively favoring the benefits of illegal actions: a highly materialistic consumer is more likely to tolerate unethical actions if they enhance personal material possessions or reduce the material possessions of others (Chowdhury and Fernando, 2013). This association implies that materialism might increase corrupt intention.

Based on the above arguments, we hypothesize that *materialism mediates the negative effect of self-esteem on corrupt intention (Hypothesis 2).*

In current research, we conducted three studies with correlated and experimental designs to test whether high self-esteem decreases corrupt intention and whether materialism plays a mediating role in the relationship between self-esteem and corrupt intention. In Study 1, we used questionnaires to investigate the correlated relationship among self-esteem, materialism, and corrupt intention. In Study 2, we manipulated self-esteem to explore its causal effect on materialism and corrupt intention. In Study 3, we manipulated materialism to further explore its mediating role. This study adopted a moderation-of-process design (Spencer et al., 2005), whereby a contextual condition to interrupt (vs. not interrupt) the causal process hypothesized and explained how the independent variable relates to the dependent variable (i.e., how self-esteem affects the corrupt intention; Jacoby and Sassenberg, 2011). A stronger ground for mediation exists if the manipulated materialism meaningfully moderates the basic effect of interest. That is, if inducing materialism can attenuate the relationship between self-esteem and corrupt intention, we can conclude that it is materialism, not another variable, that accounts for the effect of self-esteem on corrupt intention.

## Study 1: Correlated Research

In Study 1, we aimed to identify direct associative evidence for the possible relationship among self-esteem, materialism, and corrupt intention. We predicted that self-esteem would be negatively correlated with corrupt intention and that materialism would mediate this correlation.

### Method

#### Ethics Statement

The study was reviewed and approved by the Committee of Protection of Subjects at Beijing Normal University. All participants provided written informed consent before the study and were debriefed at the end of the research according to the established committee guidelines. This procedure was also followed in Studies 2 and 3.

#### Participants

We recruited 462 participants (265 women, 197 men) from two universities in China. The participants were between the ages of 17 and 22 (*M* = 18.73 years, *SD* = 1.11). To ensure the diversity of the sample, we included participants from different majors such as biology, accounting, information technology, education, and the arts.

#### Materials

##### Self-esteem

The widely recognized Chinese version of the 10-item Rosenberg Self-Esteem Scale (Rosenberg, 1965; Li et al., 2011) was used to measure participants’ self-esteem. One example item was “I feel that I’m a person of worth, at least on an equal plane with others.” The participants were instructed to indicate their agreement with each statement on a 7-point Likert scale (1 = strongly disagree, 7 = strongly agree). The self-esteem index was calculated as the average score of these 10 items, with higher scores representing higher self-esteem (*Cronbach’s* α = 0.824).

##### Materialism

The widely recognized Chinese version of the 18-item Material Values Scale (MVS; Richins and Dawson, 1992; Li and Guo, 2009) was used to measure participants’ materialism. One example item was “I admire people who own expensive homes, cars, and clothes.” The participants were instructed to indicate their agreement with each statement on a 7-point Likert scale (1 = strongly disagree, 7 = strongly agree). The materialism indicator was calculated as the average score of these 18 items, and higher scores represented a higher level of materialism (*Cronbach’s* α = 0.802).

##### Corrupt intention

A 14-item corrupt intention measure (Leong and Lin, 2009) was adapted to measure participants’ corrupt intention. We changed a few wordings of the original scale to assess the personal intention to engage in corruptive behavior. For example, the original item “Business corruption is inevitable in some cultures” was adapted by deleting “in some cultures”; the original item “When dealing with a business partner from abroad, it is important to inform the relevant authorities if the overseas partner asks for a bribe (R)” was adapted by deleting “from abroad” and “overseas”; the original item “In some countries, it is alright to pay someone extra in order to get things done quickly even if the law forbids such practices” was adapted by using “In some situations” to replace “In some countries.” The measure was translated into Chinese and back-translated for accuracy by a Chinese–English bilingual speaker. Participants were instructed to indicate their agreement with each statement on a 9-point Likert scale from 1 (“completely disagree”) to 9 (“completely agree”). The average score of these 14 items was calculated as a corrupt intention index, with a higher rating representing a stronger corrupt intention (*Cronbach’s* α = 0.773).

#### Procedure

After providing informed consent, the participants completed an online survey, including several questionnaires, in a computer room. These questionnaires included the self-esteem scale, the materialism measure, the corrupt intention measure, and other unrelated measures to prevent the participants from guessing the purposes of the research. After the participants completed the questionnaires, they were asked to provide their demographic information, including their sex, age, major, and birthplace. Finally, the participants were debriefed.

### Results and Discussion

#### Preliminary Analyses

The correlations between the three variables, the means, and the standard deviations are shown in **Table 1.** The results demonstrated that self-esteem was negatively associated with corrupt intention (*r* = –0.181, *p* < 0.001), which supported Hypothesis 1.

**Table 1:** Means, standard deviations, and correlation matrix among all variables.

Variables	Mean	*SD*	1	2
(1) Self-esteem	4.82	0.92		
(2) Materialism	3.61	0.81	–0.264^∗∗^	
(3) Corrupt intention	4.06	1.16	–0.181^∗∗^	0.434^∗∗^

*^∗∗^p < 0.01.*

Additionally, we observed that each participant’s gender and age had nearly significant correlations with corrupt intention, which was consistent with previous research indicating that demographic variables, such as age and gender, are related to moral reasoning (Haidt et al., 1993) and corrupt intention (Kennedy and Kray, 2014; Tan et al., 2016b). Thus, we subsequently included the covariance paths for age and gender in our mediation analyses.

#### Mediation via Materialism

**FIGURE 1:**
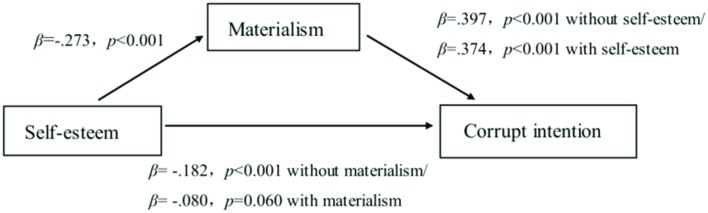
**Mediating model in Study 1 with controls including age and gender**.

We then tested our prediction that materialism mediates the association between self-esteem and corrupt intention using bootstrapping procedures (Preacher and Hayes, 2004). The analyses showed that self-esteem significantly predicted corrupt intention (β = –0.182, *SE* = 0.056, *t* = –4.127, *p* < 0.001). The variations in materialism predicted by self-esteem (a; β = –0.273, *SE* = 0.040, *t* = –6.048, *p* < 0.001) and the variations in corrupt intention predicted by materialism (b; β = 0.397, *SE* = 0.059, *t* = 9.582, *p* < 0.001) were both significant. After controlling for the effect of materialism, the direct effect of self-esteem on corrupt intention became non-significant (β = –0.080, *SE* = 0.054, *t* = –1.89, *p* = 0.060). A bootstrapping procedure comprising 5,000 samples provided additional evidence that the 95% confidence interval for the direct effect of self-esteem was [–0.207, 0.004], including zero, whereas the indirect effect was [–0.198, –0.073], not including zero (see **Figure 1**). These results support Hypothesis 2 that materialism accounts completely for the association between self-esteem and corrupt intention.

## Study 2: Causal Research

Although Study 1 confirmed a negative association between self-esteem and corrupt intention, as well as the mediating role of materialism, it inadequately established a rigorous causal relationship. Potentially, high self-esteem decreases corrupt intention, but the opposite could also be plausible. To overcome this limitation, in Study 2, self-esteem was manipulated by random feedback on a personality test, thus placing participants into either a high or a control condition. We also extended the previous results by expanding on responses to a business scenario, which consisted of a behavior with real consequences. We expected that when self-esteem increased, individuals would exhibit lower materialism and a lower corrupt intention.

### Method

#### Participants

One hundred participants (35 women, 64 men, 1 unreported) were recruited at a Chinese university through the campus network. The participants spanned the ages of 17 to 21 (*M* = 19.09 years, *SD* = 0.84). To ensure the diversity of the sample, participants from different majors were included. They were randomly assigned to one of two experimental conditions: the high self-esteem condition (*n* = 44) or the control condition (*n* = 56).

#### Materials

##### Manipulation of self-esteem

We adopted the research paradigm of Aronson and Mettee (1968) to manipulate self-esteem. The participants were asked to complete a shortened version of the California Personality Inventory (CPI) to evaluate their personalities. This version contained only 25 items from the six scales related to self-esteem. However, the primary experimental purpose of this test was merely to provide the opportunity and rationale for situationally manipulating the participants’ self-esteem via pre-programmed feedback regarding the participants’ personality test results. We emphasized that the computer would score the personality inventories through a complicating coding process and then provide feedback. The participants in the high self-esteem condition received the following feedback (Aronson and Mettee, 1968; Greenberg et al., 1992):

“The profile indicates you have a stable personality and are not given to pronounced mood fluctuations of excitement or depression. Your stableness does not seem to reflect compulsive tendencies, but rather an ability to remain calm and level-headed in almost any circumstance. You are straightforward when making a decision, and never punctilious. You have strong heart. Any negative evaluations from others cannot threaten your sense of value. You are mentally mature for your age, and remain so going forward.”

Participants in the control condition received the following feedback:

“The profile indicates you have a fairly stable personality, but occasionally experience mood fluctuations of excitement or depression. Your stableness reflects your aspirations for freedom and independence from others, but it may be a bit unrealistic. Your profile suggests that you might be very careful but meticulous when making unimportant decisions. You have fairly strong heart, but you need to be more mature going forward.”

To check the effectiveness of the manipulation, the participants were presented with a 10-item Rosenberg Self-Esteem Scale (Rosenberg, 1965), as used in Study 1, on the computer (*Cronbach’s* α = 0.645).

##### Materialism

We administered the same materialism scale as used in Study 1 to measure participants’ level of materialism (*Cronbach’s* α = 0.821).

##### Corrupt intention

A business corruption scenario (Mazar and Aggarwal, 2011) was adapted to determine the participants’ corrupt intentions. Participants were assumed the role of a sales agent who had to compete against two other firms to win a contract from an international buyer and earn a commission. The sales agent was contemplating whether to offer an unofficial payment (bribe) to the potential international buyer to help win this contract. It was translated into Chinese and back-translated for accuracy by a Chinese–English bilingual speaker. After reading the scenario, the participants were asked to answer the following five questions (Huang et al., 2015): “As for me, the way of giving the money is not in my mind (R),” “If not taking other factors into consideration, I am willing to give the money,” “I never consider giving the money (R),” “If I have the same situation to face in the future, I will still give the money,” and “I think I will give the money to him.” The responses to these items were scored on a 9-point Likert scale from 1 (“completely disagree”) to 9 (“completely agree”). The average score of these five items was calculated as a corrupt intention index, with higher ratings representing higher corrupt intentions (*Cronbach’s* α = 0.791).

#### Procedure

All participants entered the laboratory and were informed that they were participating in a study concerned with the correlation between personality test scores and social behavior. The participants initially completed the CPI to evaluate their personalities and received random feedback, which randomly divided them into two conditions: the high self-esteem condition or the control condition. After receiving the random feedback (positive or neutral) regarding their personalities, the participants were instructed to complete the next questionnaire. At the end of the study, the participants were thanked with stationery gifts and debriefed.

### Results and Discussion

#### Manipulation Check

As expected, participants in the high self-esteem condition had significantly higher self-esteem scores (*M* = 4.76, *SD* = 0.59) than those in the control condition did (*M* = 4.48, *SD* = 0.68), *t*(98) = 2.18, *p* < 0.05, *Cohen’s D* = 0.28. This finding suggests that the manipulation was effective.

#### Preliminary Analyses

As expected, the manipulated self-esteem was significantly correlated with materialism (*r* = –0.276, *p* < 0.01) and corrupt intention (*r* = –0.235, *p* < 0.05). Furthermore, the mean materialism score of participants in the high self-esteem condition (*M* = 4.47, *SD* = 1.02) was significantly lower than that of the control condition (*M* = 5.02, *SD* = 0.90), *t*(98) = –2.85, *p* < 0.01, *Cohen’s D* = –0.57. The mean corrupt intention score of the high self-esteem condition (*M* = 3.80, *SD* = 1.44) was significantly lower than that of the control condition (*M* = 4.56, *SD* = 1.69), *t*(98) = –2.39, *p* < 0.05, *Cohen’s D* = –0.49. These results suggest that increased self-esteem had a negative effect on materialism and corrupt intention.

#### Mediation via Materialism

We then coded the high self-esteem and control conditions as +1 and 0, respectively, and further explored the mediating effect of materialism through bootstrapping procedures (Preacher and Hayes, 2004). The analyses showed that the self-esteem condition significantly predicted corrupt intention (β = –0.235, *SE* = 0.319, *t* = –2.39, *p* = 0.019). The variations in materialism predicted by the self-esteem condition (a; β = –0.276, *SE* = 0.192, *t* = –2.85, *p* = 0.005) and the variations in corrupt intention predicted by materialism (b; β = 0.438, *SE* = 0.149*, t* = 4.823, *p* < 0.001) were both significant. After controlling for the effect of materialism, the direct effect of the self-esteem condition on corrupt intention became non-significant (β = –0.123, *SE* = 0.306, *t* = –1.31, *p* = 0.193). A bootstrapping procedure with 5,000 samples provided additional evidence that the 95% *CI* for an indirect effect of self-esteem on corrupt intention through materialism was [–0.763, –0.125], not including zero. The direct effect was [–1.008, 0.207], including zero, which implies that materialism completely mediated the effect of self-esteem on corrupt intention.

**FIGURE 2:**
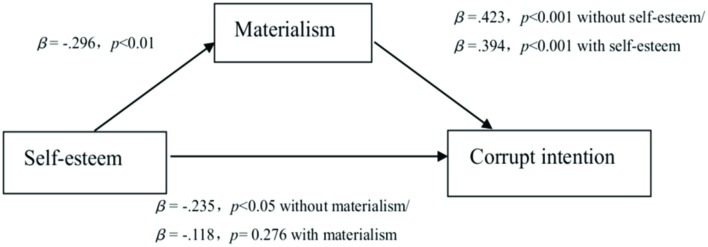
**Mediating model in Study 2 with controls including age and gender**.

The results were identical to the findings of the analysis with controlled variables including age and gender (see **Figure 2**). The analyses showed that the self-esteem condition significantly predicted corrupt intention (β = –0.235, *SE* = 0.370, *t* = –2.091, *p* = 0.039). The variations in materialism predicted by the self-esteem condition (a; β = –0.296, *SE* = 0.216, *t* = –2.677, *p* = 0.009) and the variations in corrupt intention predicted by materialism (b; β = 0.423, *SE* = 0.161*, t* = 4.438, *p* < 0.001) were both significant. After controlling for the effect of materialism, the direct effect of the self-esteem condition on corrupt intention became non-significant (β = –0.118, *SE* = 0.356, *t* = –1.096, *p* = 0.276). A bootstrapping procedure indicated that the 95% *CI* for an indirect association between the self-esteem condition and corrupt intention operating through materialism with the controlled variables was [–0.818, –0.096], not including zero. The direct effect was [–1.099, 0.317], including zero, which implies materialism completely mediated the effect of self-esteem on corrupt intention.

The results showed that high self-esteem decreased the corrupt intention and that the buffered materialism mediated the relationship, thus further supporting our hypotheses. In other words, the results indicated that individuals whose self-esteem was enhanced would be less obsessed with material desires, thus were less likely to commit corrupt acts. Together, these results bolster our theoretical framework, thereby indicating that increasing self-esteem decreases the corrupt intention and that materialism helps explain this effect.

## Study 3: Psychological Process Examining

Although Study 2 confirmed the causal link that high self-esteem decreased materialism and then buffered corrupt intention, the mediator accounting for the relationship between self-esteem and corrupt intention was essentially correlated. It is possible that evidence of mediation was obtained spuriously because of the relation between the measured mediator and the true psychological process. To confirm that the relationship between self-esteem and corrupt intention was influenced by materialism, in Study 3, we adopted the “moderation-of-process” design (Spencer et al., 2005) to further examine the psychological process. We primed materialism and predicted that the negative association between self-esteem and corrupt intention, as in Studies 1 and 2, would be reduced or eliminated when materialism was elicited. We expected that the differences in the degree of materialism could explain why individuals with varying self-esteem levels differ in their corrupt intention.

### Method

#### Participants

A total of 127 participants (101 women, 25 men, 1 unreported) were recruited at a Chinese university. The participants spanned the ages of 18 to 27 (*M* = 20.75 years, *SD* = 2.29). They were randomly assigned to one of two experimental conditions: the materialism-induction condition (*n* = 63) or the control condition (*n* = 64).

#### Materials

##### Self-esteem

A 10-item Rosenberg Self-Esteem Scale was used, as in Study 1, to measure the participants’ self-esteem levels (*Cronbach’s* α = 0.817).

##### Manipulation of materialism

To prime materialism, we relied on and adapted from a common experimental procedure, the scrambled-sentences task (Srull and Wyer, 1979; Bauer et al., 2012). The participants were presented with 30 word strings, each consisting of five words. For each string, the participants were instructed to select and order four of the words to form a valid Chinese sentence. For participants randomly assigned to the materialistic-cue condition, 20 of these word strings (67%) contained a word related to materialistic concepts (e.g., *buy, status, asset, and expensive*). For example, participants in this condition were asked to construct sentences out of such strings as “expensive, his, was, everybody, watch.” In the control condition, highly similar word sets were created except that in each instance, materialistic concepts were replaced with mundane, non-materialistic ones (e.g., replacing the word *expensive* with the word *accurate*). Correspondingly, participants in this condition were presented with such neutral strings as “accurate, his, was, everybody, watch.”

To check the effectiveness of the manipulation, the participants were asked to complete the identical materialism scale as in Study 1. We only changed prolonged words such as “always” in the original scale into present tense words such as “now” (*Cronbach’s* α = 0.770).

##### Corrupt intention

A business corruption scenario was used, as in Study 2, and five questions were asked to measure the participants’ corrupt intention (*Cronbach’s* α = 0.867).

#### Procedure

After providing informed consent, the participants completed several experimental tasks on paper in a private cubicle. The first task was presented as a study of the “cognitive aspects of linguistic processing.” Participants were asked to select and order four of the words to form a valid Chinese sentence. In reality, this was the priming task. Next, the participants completed ostensibly unrelated questionnaires. Finally, the participants were thanked, debriefed and paid RMBaaa10 each.

### Results and Discussion

#### Manipulation Check

As expected, the participants in the materialism-induction condition exhibited a higher materialistic tendency (*M* = 3.80, *SD* = 0.65) than the participants did in the control condition (*M* = 3.57, *SD* = 0.62), *t*(125) = 2.06, *p* < 0.05, *Cohen’s D* = 0.37. This result suggests that the manipulation of materialism was effective. However, there was no difference in self-esteem between the materialism-induction (*M* = 4.97, *SD* = 0.82) and the control condition (*M* = 4.99, *SD* = 0.83), *t*(125) = 0.10, *p* = 0.92, suggesting that materialism did not influence self-esteem.

#### Test of Interaction

We predicted that the experimental condition would moderate the association between self-esteem and corrupt intention such that the negative relationship would be present in the control condition, but not in the materialism-induction condition. To test this prediction, we regressed corrupt intention with self-esteem, the experimental condition (materialism-induction vs. control), and the interaction of self-esteem and the experimental condition. To interpret the results, we centered self-esteem prior to the analysis because it is a continuous variable (Aiken et al., 1991).

**FIGURE 3:**
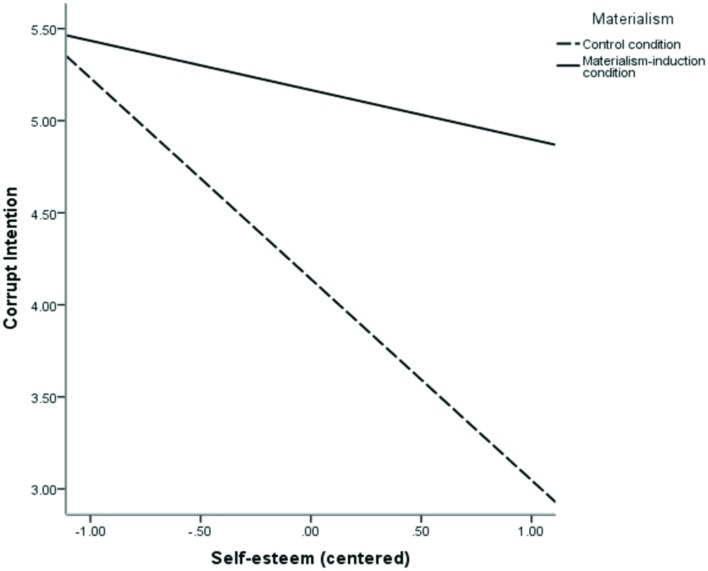
**Corrupt intention as a function of self-esteem and materialism in Study 3**.

The results are presented in **Table 2.** After controlling for gender and age, self-esteem was negatively associated with corrupt intention. This association was qualified by a significant interaction between self-esteem and experimental condition (*p* = 0.043), displayed in **Figure 3.** To interpret the interaction, we tested the simple slopes using the procedures described by Aiken et al. (1991). In the control condition, the simple slope of self-esteem on corrupt intention was significant: *simple slope* = –1.09*2, SE* = 0.294, *t*(120) = –3.716, *p* < 0.001, a finding consistent with Studies 1 and 2. By contrast, and consistent with our prediction, in the materialism-induction condition: *simple slope* = –0.268*, SE* = 0.296, *t*(120) = –0.906*, p* = 0.367. When primed to a materialistic mindset, the corrupt intention of participants with higher self-esteem was comparable to participants with lower self-esteem. This finding suggests that lower self-esteem individuals tended to favor corrupt behavior, at least partly because they experience a higher level of materialism than individuals with higher self-esteem do.

**Table 2:** Regression results predicting corrupt intention in Study 3.

	Model 1				Model 2			
Variable	*B*	*SE*	β	*t*	*B*	*SE*	β	*t*
Gender					–0.408	0.419	–0.084	–0.974
Age					0.090	0.075	0.106	1.204
Self-esteem	–0.611	0.195	–0.257	–3.14^∗∗^	–0.680	0.215	–0.283	–3.16^∗∗^
Condition (1 = materialism, –1 = control)	0.498	0.159	0.257	3.14^∗∗^	0.509	0.160	0.261	3.19^∗∗^
Self-esteem × Condition	0.487	0.195	1.265	2.497^∗^	0.412	0.202	1.070	2.043^∗^
		
	*R*^2^ = 0.176 *F*(3,123) = 8.780^∗∗^				*R*^2^ = 0.196 *F*(5,120) = 5.862^∗∗^			

*^∗^p < 0.05, ^∗∗^p < 0.01.*

In Study 3, we adopted the moderation-of-process design and observed that the negative effect of self-esteem on corrupt intention observed in Studies 1 and 2 was reduced when materialism was experimentally induced. The results suggest that one reason that individuals with high self-esteem tend to have lower corrupt intentions is that they tend to have a lower passion for materialism. Further, corruption might serve as a coping pattern accompanied with high materialism to satisfy the needs of individuals lacking self-esteem. In sum, the results imply that increased self-esteem decreases corrupt intention through a lower level of materialism.

## General Discussion

The present three studies showed that high self-esteem decreased individuals’ tendencies toward corruption and that materialism mediated this relationship based on a sample of Chinese university students. We confirmed our hypotheses in three studies using different measures of corrupt intention and different strategies to examine the process. The results of Study 1 suggest that self-esteem was negatively associated with the corrupt intention and that materialism mediated this relationship. In Study 2, we manipulated self-esteem, and the results indicated that increased self-esteem caused a low level of materialism and corrupt intention. In Study 3, we used a different strategy to examine the underlying psychological process, determining that the differences in the degree of materialism explain why individuals with varying self-esteem levels differ in their corrupt intention. These studies converged to show that increased self-esteem caused a low level of materialism, which in turn decreased corrupt intention.

The findings from the present research make a significant contribution to the literature of self-esteem. Previous research has shown that high self-esteem tends to be associated with, albeit might not cause, positive outcomes, such as better performance (Kuster et al., 2013), interpersonal success (Sommer and Baumeister, 2002), or health and well-being (Harter, 1999); whereas low self-esteem tends to be associated with problems such as cheat at a game (Aronson and Mettee, 1968), cheat on exams (Iyer and Eastman, 2006), and other dishonest behaviors (Graf, 1971). Our research expands the study of self-esteem to the field of corruption. To the best of our knowledge, our results provide the first empirical evidence that high self-esteem decreases corrupt intention. Corruption impels individuals not only to compromise their ethical standards for their own benefit, but also to violate the law (Gupta et al., 2002; UN, 2003; Uslaner, 2008). Our findings imply that to maintain, enhance, and protect individuals’ self-esteem is such a powerful motivation (Baumeister, 1998) that it drives individuals to commit corrupt acts with the heavy cost.

Furthermore, the present research also contributes to the effects of self-esteem on unethical behaviors like corruption, by identifying materialism as an underlying psychological process. The mediating role of materialism explains why individuals endorse less corruption after self-esteem is enhanced. On the one hand, the increasing self-esteem depresses materialism and corrupt intention. Material possessions are important for maintaining a self-concept (Belk, 1985) and are instruments for coping with doubts regarding self-worth or competence (Chang and Arkin, 2002; Jiang et al., 2015). Thus, when self-esteem is enhanced, the pursuit of material pleasures by means of corruption, which can temporarily produce prestige and wealth, is unnecessary, as inferred from the results of Study 2. On the other hand, the results indicate that materialism could buffer the negative relationship between self-esteem and corrupt intention. When primed to a materialistic mindset, the corrupt intention of individuals with higher self-esteem was comparable to individuals with lower self-esteem. Apart from coping with doubts concerning self-worth, materialism might also serve as a justification allowing individuals with higher self-esteem to commit corruption. This justification reduces the ethical dissonance, which represents the tension between moral-self and unethical behavior (Barkan et al., 2015). It could also be inferred from Study 3 that, if the pursuit of material decreases, individuals will be prone to commit less corrupt acts regardless of their self-esteem level, which would be very critical to control corruption.

The present research adds to the current debate on whether material possession amounts to self-worth. Previous research has found that an individual’s self-esteem is negatively associated with materialism (Jiang et al., 2015), and that the relational change between the two variables over time has been demonstrated by further direct evidence (Yurchisin and Johnson, 2004; Chaplin and John, 2005; Isaksen and Roper, 2012). These results suggest that materialistic values could be used to cope with uncertainty about self-worth or competence (Chang and Arkin, 2002; Kasser, 2002; Jiang et al., 2015), thus might buffer threatened self-esteem. However, this function has only been observed as a temporary method to cope with the suffering of low self-esteem and might actually reduce an individual’s well-being in the long term (Burroughs and Rindfleisch, 2002; Kasser et al., 2014). In fact, possession cannot amount to self-worth because increasing self-esteem increases subjective well-being and life satisfaction (Diener et al., 1995; Schimmack and Diener, 2003). In contrast, an over-emphasis of materialistic goals might augment negative emotions and depressive symptomatology (Kasser and Ryan, 1993; Kashdan and Breen, 2007), inhibit positive emotion and positive social relationships, hinder socialization, inflict losses on subjective well-being, and undermine life satisfaction (Diener and Biswas-Diener, 2002; Christopher et al., 2007; Jiang et al., 2015). In this regard, our Study 3 also showed that increasing materialism did not enhance self-esteem.

The results from the current research have practical implications for anti-corruption. For China, rapid economic growth has introduced multiple pressures and temptations, including status, riches, and fame. Lü (2000) shows that three common types of corruption exist concerning Chinese people’s daily life: graft, rent-seeking, and prebendalism. The main thread linking these different types of corruption is that many individuals regard public office as a business (Van Klaveren, 1989; Tan et al., 2016a), at the expense of morality for status and possessions, which thus become measures of personal success and self-worth. Individuals with positive self-esteem may inhibit their sensitivity to external material possessions (Richins, 1999; Kasser and Kasser, 2001) and are less likely to engage in corrupt behaviors. For individuals lacking positive self-esteem, materialism may be controlled by removing materialistic messages regarding money, possessions, status, and image from the environment, by providing them with strategies to reduce the effect of those messages when they are encountered, or by exposing them to scenes or objectives that reflect nature (Weinstein et al., 2009; Kasser, 2016). Such suppression of materialism may decrease the incidence of corrupt behaviors. Additionally, by extensively exposing individuals to prevailing ethical norms (Gong and Wang, 2013; Tan et al., 2016a) and information on the potential long-term damage of materialism and corruption, they may determine that the costs likely outweigh the benefits and become less engrossed in corruption to achieve wealth.

Some limitations of the current research should be mentioned. First, it seems that receiving a positive feedback could potentially boost positive mood, and mood and emotion have been shown to be linked to ethics in some ways (Bazerman et al., 2011; Gino and Shea, 2012; Teper et al., 2015). However, we did not measure the participants’ mood in Study 2. Future study should measure and thereby statistically control mood when manipulating self-esteem to further explore its effect on corruption. Second, also in Study 2, we explored the effect of self-esteem on corrupt intention by randomly assigning the participants to either high self-esteem condition or control condition. Unfortunately, we failed to successfully manipulate low self-esteem following the same paradigm of Aronson and Mettee (1968). Future study could use a different paradigm to manipulate low self-esteem for further exploring its effect. Third, only self-reported measures were used in current research to capture corrupt intention. However, past researchers have noted that the relationship between predictor variables and unethical intentions is often weaker than the relationship between predictor variables and actual unethical behavior (Kish-Gephart et al., 2010). Therefore, future study should further explore the effect of self-esteem on corrupt behavior by using the method of bribery game (Abbink and Hennig-Schmidt, 2006; Huang et al., 2015).

## Author Contributions

YL contributed to all aspects of work for this article. LL contributed to conception, design, and revising the article critically. XT contributed to data collection, design, and interpretation. ZH and JD contributed to data analysis, interpretation, and revising the article critically. WZ contributed to interpretation and revising the article carefully.

## Conflict of Interest Statement

The authors declare that the research was conducted in the absence of any commercial or financial relationships that could be construed as a potential conflict of interest.
